# Ophthalmic Manifestations in A Patient With Kabuki Syndrome: A Case Report With a KDM6A Gene Variant

**DOI:** 10.1155/crop/1626256

**Published:** 2026-09-15

**Authors:** Tülin Öğreden, Gülçiçek Cayhan

**Affiliations:** ^1^ University of Health Sciences, Ophthalmology Department, Başakşehir Çam and Sakura City Hospital, Istanbul, Turkey, uhsr.ac.in

**Keywords:** electrophysiology, facial dysmorphology, Kabuki syndrome, myopia, optic disc hypoplasia

## Abstract

Kabuki syndrome (KS) is a rare genetic disorder with a wide phenotypic spectrum and several genotypic variants. KS can result from mutations on Chromosome 12 (KMT2D gene) and Chromosome X (KDM6A gene). The KDM6A gene mutation is seen in approximately 2%–6% of Kabuki syndrome cases. In this case report, we present a patient with KS who had a rare and potentially pathogenic heterozygous c.3457dup (p.His153ProfsTer2) in the KDM6A gene from an ophthalmological perspective. Examination revealed distinctive facial features and some common and rare ophthalmological findings. A small number of cases have reported high myopic refractive error and optic disc hypoplasia, as in this case.

## 1. Introduction

Kabuki syndrome (KS), initially characterized by Kuroki et al. [1], is a congenital malformation and neurodevelopmental disorder. The term “Kabuki” is derived from the distinctive facial features reminiscent of the makeup used in traditional Japanese kabuki theater. The phenotypic spectrum of KS includes facial dysmorphism, neurological impairments, postnatal growth retardation, skeletal and limb anomalies, cardiovascular and urogenital abnormalities, cleft palate, microretrognathia, malocclusion, hearing impairment, and a range of visceral malformations [1]. The specific manifestations and their severity vary widely among affected individuals.

Because of the rarity of KS, data regarding ophthalmologic involvement remain limited. The most frequently reported ophthalmological abnormalities are strabismus and eyelid developmental anomalies, occurring in approximately 20.5% and 14.4% of cases, respectively [2]. Other reported findings include microphthalmia, microcornea, corneal opacities and pannus, blue sclerae, cataracts, nasolacrimal duct obstruction, caruncular lipoma, retinal telangiectasia, retinal pigmentary changes, and nystagmus [3]. Less frequently observed findings include colobomatous microphthalmia and dysplastic optic discs. Data on refractive errors in KS are limited and heterogeneous.

KS is genetically heterogeneous and is most commonly associated with pathogenic variants in KMT2D (KS1) or KDM6A (KS2). In this case report, we present a patient with KS, clinically diagnosed and supported by a likely pathogenic KDM6A (KS2) variant. The patient exhibits significant ophthalmologic findings, including high refractive errors.

## 2. Case Report

**Figure 1:**
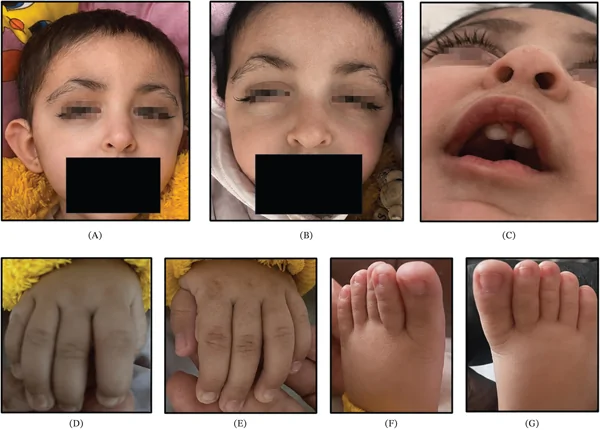
(A) Cranial asymmetry, microcephaly, broad nasal bridge, long palpebral fissures, arched eyebrows, eversion of the lateral third of the lower eyelid, and an antimongoloid slant. (B) Arched eyebrows, lagophthalmos, and elongated eyelashes. (C) High‐arched and incomplete cleft palate with dental misalignment and long eyelashes. (D, E) Long, slender fingers and clinodactyly of the fifth digits. (F, G) Long, thin toes and indistinct toe pads.

For this case report, written and verbal informed consent was obtained from the legal guardians of patient. A 15‐month‐old female presented with complaints of excessive tearing. No relevant features were identified in the family history. Systemic assessment demonstrated developmental delay and some facial features, including a broad nasal bridge, long palpebral fissures, arched eyebrows, craniosynostosis, microcephaly, a high‐arched palate, and an incomplete cleft palate (Figure 1A–C). Cranial asymmetry, long and slender digits, prominent finger pads, and clinodactyly of the fifth fingers also were noted (Figure 1D–G).

She has hypotonia, with no history of seizures or epilepsy. Neuroimaging demonstrated agenesis of the corpus callosum.

Ophthalmic examination revealed eversion of the lateral third of the lower eyelid and elongated eyelashes (Figure 1A–C), as well as tear overflow, associated with eyelid contour abnormalities.

She has horizontal pendular nystagmus. Visual evoked potential (VEP) testing showed prolonged P100 wave latency and reduced amplitude.

The corneal diameter measured 10 mm in both meridians. Intraocular pressure was normal. The sclera was thin and bluish. Both the upper and lower eyelids exhibited lagophthalmos with lamellar insufficiency (Figure 1B). Bilateral exposure keratopathy was noted.

**Figure 2:**
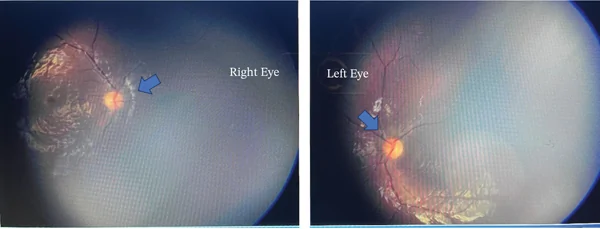
Fundus photograph. The blue arrow indicates a hypoplastic optic nerve, whereas the macular reflex appears normal.

Cycloplegic refractions were −6.25 −3.00 × 120° in the right eye and −6.00 −1.75 × 114° in the left eye. Corneal curvatures were K1: 46.75 D and K2: 49.75 D in the right and K1: 45.75 D and K2: 47.50 D in the left eye. Axial length could not be measured because of equipment limitations. Refractive correction was applied. Fundus examination revealed a small, pale optic disc (Figure 2).

Ophthalmic findings in our case are summarized in Table 1, with particular emphasis on comparison with the more common KMT2D‐related variant.

**Table 1:** Ocular findings in KDM6A‐related Kabuki syndrome: Comparison of the present case with previously reported cases.

Ocular findings	KMT2D‐related KS^a^	KDM6A‐related KS^b^	Present case
Long palpebral fissures	Common	Moderately common	Present
Eversion of lower eyelid (lateral)	Common	Limited reports	Present
Epiphora due to eyelid anomaly and lagophthalmos	Limited reports	Limited reports	Present
Ptosis	Limited reports	Limited reports	Absent
Strabismus	Moderately common	Moderately common	Absent
Nystagmus	Moderately common	Rare	Present
Refractive errors	Common	Limited reports	High myopic astigmatism
Optic disc anomalies	Rare	Limited reports	Hypoplastic, pale disc
Coloboma	Rare	Limited reports	Absent
Microphthalmia	Rare	Limited reports	Absent
Corneal abnormalities	Rare	Limited reports	Steep cornea

^a^Kabuki syndrome associated with pathogenic variants in the KMT2D (lysine methyltransferase 2D) or KS1.

^b^Kabuki syndrome associated with pathogenic variants in the KDM6A gene (lysine demethylase 6A) or KS2.

Findings were classified as common (> 50%), moderately common (10%–50%), or rare (< 10%); fewer than three reports were defined as limited.

Whole‐exome sequencing was performed on genomic DNA obtained from peripheral blood, and data were analyzed using a standard bioinformatics pipeline including alignment, variant calling, and annotation. A heterozygous frameshift variant in the X‐linked KDM6A gene (c.3457dup, p.His153ProfsTer2) was identified. This variant was absent from population (gnomAD) and disease‐associated (ClinVar) databases and was classified as likely pathogenic according to ACMG criteria based on PVS1 (predicted loss‐of‐function) and PM2 (absence from population databases), supporting its potential contribution to the observed phenotype. The KDM6A gene encodes a histone demethylase involved in epigenetic regulation, and its dysfunction is considered relevant to disease pathogenesis.

## 3. Discussion

KS is a rare multisystem developmental disorder that has been reported in diverse ethnic populations worldwide, although its prevalence has been best characterized in Japan, where it is estimated to affect approximately 1 in 32,000 individuals [4]. Cases have also been documented in Northern Europe, Brazil, Vietnam, the Philippines, the East Indies, China, Mexico, and across Arab and African regions [4]. In contrast, epidemiological data from Turkey remain limited, with only a small number of published cases available to date [5, 6]. This limited representation makes it difficult to define the full clinical spectrum of KS in underrepresented populations and underscores the value of reporting well‐characterized cases.

Because of the phenotypic and genotypic heterogeneity of KS, international consensus diagnostic criteria were established by Adam et al. [7]. According to Adam et al. [7], the diagnosis requires hypotonia, developmental delay, and/or intellectual disability, together with either a pathogenic variant in KMT2D (KS1) or KDM6A (KS2) or the presence of characteristic dysmorphic facial features, including long palpebral fissures with eversion of the lateral lower eyelid and associated craniofacial findings. Our patient fulfilled these consensus criteria, and molecular confirmation supported the clinical diagnosis.

Between 55% and 80% of KS cases are attributable to pathogenic variants in the KMT2D gene, which encodes a histone methyltransferase protein that plays a pivotal role in the regulation of gene expression through chromatin remodeling. According to Boniel et al. [8], KDM6A, identified in approximately 5%–8% of patients with KS, encodes lysine‐specific histone demethylase 6A, an enzyme involved in epigenetic regulation through histone modification and chromatin remodeling. KDM6A gene variations have been reported in a relatively limited number of cases, as described in previous studies [9–11]. The identification of a KDM6A variant in our patient, therefore, represents a relatively uncommon molecular subtype of KS.

Both KMT2D and KDM6A regulate chemical modifications on histone proteins, which are critical for chromatin organization. KMT2D promotes transcriptional activation, whereas KDM6A relieves transcriptional repression through histone modification; together, these proteins play complementary roles in the regulation of gene expression during development. Although disruption of either mechanism leads to dysregulated gene expression during neurodevelopment, these distinct molecular functions have been proposed to contribute to the largely overlapping yet variably expressed neurological and ocular features observed in KS1 and KS2. In the present case, a potentially pathogenic variant in the KDM6A gene was identified, representing a rare variant associated with KS2 [9–11]. Given the limited number of reported KDM6A‐associated cases, detailed clinical descriptions remain valuable for improving our understanding of the ophthalmic manifestations of KS2.

Kabuki syndrome Type 1 (KS1) is most often sporadic and caused by de novo mutations, whereas Type 2 (KS2) is inherited in an X‐linked manner. Patients with KDM6A mutations show a broad phenotypic spectrum ranging from typical KS to milder presentations [9, 11].

Banka et al. [11] reported that infants with KS2 commonly present with hypotonia and feeding difficulties. Poor postnatal growth and short stature are also frequent clinical features, with short stature being particularly characteristic of KS2. Developmental delay and learning difficulties are commonly observed in affected females and are generally mild to moderate in severity [11]. In our patient, hypotonia and developmental delay were consistent with the clinical features. She also exhibited craniosynostosis, a high‐arched palate with an incomplete cleft palate, and long slender fingers with fifth‐finger clinodactyly.

The ocular phenotype of KS2 has been reported in only a limited number of studies. The available evidence suggests that it largely overlaps with that of KS1 and includes long palpebral fissures, eversion of the lateral third of the lower eyelid, ptosis, and strabismus. In our patient, long palpebral fissures and eversion of the lateral third of the lower eyelid were present, whereas ptosis and strabismus were absent.

In the study by Faundes et al. [12], ophthalmologic abnormalities were reported in 58.3% of patients with KS2, with strabismus observed in 31.3% and nystagmus in 10.4% of cases. Previous studies in KS cohorts have reported strabismus rates ranging from 38% to 42%, suggesting that strabismus frequency in KS2 appears generally comparable with that reported in KS1 cases [13].

In our patient, fundus examination revealed a hypoplastic and pale optic disc, adding to the limited reports of optic nerve abnormalities in KS2. A previous case series described optic disc coloboma in one patient and a dysplastic, elevated optic disc in another [14]. Our patient also had hypotonia without a history of seizures or epilepsy, and neuroimaging demonstrated agenesis of the corpus callosum. Structural abnormalities such as corpus callosum agenesis and optic disc hypoplasia have been suggested to contribute to the development of nystagmus [15]. In our patient, nystagmus may be related to underdeveloped visual pathways, as supported by VEP findings.

Eyelid abnormalities may lead to lacrimal dysfunction and exposure keratopathy in KS. In our patient, lower eyelid contour may have contributed to reflex tearing and lagophthalmos. This requires close follow‐up to prevent long‐term complications.

Refractive errors in KS patients exhibit considerable variability between different genotypes. In our case, the presence of high myopic astigmatism in both eyes is noteworthy and may represent a feature of the syndrome. Corneal topography demonstrated steep corneal curvature. There are reports of corneal involvement in KS patients, and close follow‐up for corneal pathologies is recommended [12, 15, 16].

In the present case, the presence of high myopic astigmatism, corneal steepening, eyelid abnormalities, exposure keratopathy, and optic nerve hypoplasia may contribute to the characterization of ocular findings in KDM6A‐associated KS. No significant strabismus was observed in our patient. In KDM6A, rare findings such as microphthalmia and chorioretinal coloboma have been reported; neither microphthalmia nor chorioretinal coloboma was observed in our patient.

This case demonstrates a spectrum of systemic and ophthalmologic features of KS. Clinical suspicion guided the genetic confirmation. Pediatricians and pediatric ophthalmologists should be familiar with these features to facilitate early diagnosis and intervention.

## Funding

No funding was received for this manuscript.

## Ethics Statement

Approval was obtained from the local ethics committee of our hospital and the study was conducted in accordance with the Helsinki declaration.

## Conflicts of Interest

The authors declare no conflicts of interest.

## Data Availability

The data that support the findings of this study are available on request from the corresponding author. The data are not publicly available due to privacy or ethical restrictions.
